# Metabolites Identification and Mechanism Prediction of Neobavaisoflavone In Vitro and In Vivo of Rats through UHPLC-Q-Exactive Plus Orbitrap MS Integrated Network Pharmacology

**DOI:** 10.3390/molecules27238413

**Published:** 2022-12-01

**Authors:** Linlin Li, Fan Dong, Bianli Wang, Jian Song, Huimin Zhang, Ping Wang, Feiran Wang, Yingying Yan, Xiao Zhang

**Affiliations:** 1 School of Pharmacy, Shandong University of Traditional Chinese Medicine, Jinan 250355, China; 2 Shandong Academy of Chinese Medicine, Jinan 250014, China; 3 School of Chinese Materia Medica, Beijing University of Chinese Medicine, Beijing 100105, China

**Keywords:** neobavaisoflavone, UHPLC-Q-Exactive Plus Orbitrap MS, metabolism, network pharmacology

## Abstract

Neobavaisoflavone is an important isoflavone component isolated from *Psoraleae Fructus*. It is used extensively worldwide because of its antibacterial, antioxidant, anti-inflammatory, anticancer, and anti-osteoporotic activities. However, there is no systematic and comprehensive research on the metabolism of neobavaisoflavone in vivo and in vitro. The study aimed to analyze the metabolic characteristics and mechanism of neobavaisoflavone for the first time. Firstly, biological samples were pretreated by the solid-phase extraction (SPE) method, methanol precipitation, and acetonitrile precipitation. Secondly, the samples were analyzed on UHPLC-Q-Exactive Plus Orbitrap MS. Thirdly, metabolites were tentatively identified based on retention time, parallel reaction monitoring strategy, diagnostic product ions, and neutral loss fragments. A total of 72 metabolites of neobavaisoflavone were tentatively identified, including 28 in plasma, 43 in urine, 18 in feces, six in the liver, and four in the liver microsome. The results suggested that neobavaisoflavone mainly underwent glucuronidation, sulfation, hydroxylation, methylation, cyclization, hydration, and their composite reactions in vivo and in vitro. In addition, nine active components with high bioavailability and 191 corresponding targets were predicted by the Swiss Drug Design database. The 1806 items of GO and 183 KEGG signaling pathways were enriched. These results showed that metabolites expanded the potential effects of neobavaisoflavone. The present study would provide the scientific basis for the further exploitation and application of neobavaisoflavone.

## 1. Introduction

*Psoraleae Fructus* is widely distributed in warm and sunny areas such as Sichuan, Anhui, Yunnan, Guizhou, and Guangxi in China [1], as well as India, Myanmar, and Sri Lanka [2]. Apart from various pharmacological effects, *Psoraleae Fructus* is often used for medicinal diets with meat, walnut kernels, wine, or other foods. It is not only delicious but also a warm tonic that can improve immunity [3,4]. Scholars have extracted a variety of chemical components from *Psoraleae Fructus*, such as coumarins, flavonoids, and monoterpene phenols [5]. The study of these complex components would provide help for further elucidation of clinical pharmacological effects and applications of *Psoraleae Fructus* [6]. Neobavaisoflavone is isoflavone extracted from *Psoraleae Fructus* with anti-inflammatory [7], anticancer [8], and antioxidant effects [9,10]. The researchers found that neobavaisoflavone could potently inhibit osteoclastogenesis and osteoclast functions in vivo and in vitro [11]. Some researchers also found that neobavaisoflavone was a potential whitening agent [12], in addition to the traditional pharmacological effects. Neobavaisoflavone might be widely used in whitening cosmetics in the future. Recent studies have shown that mice develop severe cholestatic liver damage after taking a certain dose of neobavaisoflavone [13,14]. Hence, the toxicity of neobavaisoflavone may manifest as liver damage. However, most of the previous studies focused on a single biological activity or pharmacological effect, and few studies systematically investigated the metabolites and mechanisms of neobavaisoflavone. Therefore, it is important to carry out a comprehensive metabolism study of neobavaisoflavone.

Metabolism refers to the biotransformation of a drug in the body. Any form of transformation and existence of drugs may be an important part of its efficacy. Therefore, metabolite identification is an indispensable step in the drug development process [15,16]. In addition, the research on the metabolism of natural products could help us to explain its pharmacological effects and predict its mechanism better [17]. At present, the combination of ultra-high-performance liquid chromatography-high-resolution mass spectrometry (UHPLC-HRMS) has been widely used for the in-depth analysis of drug metabolites [18]. UHPLC-HRMS shows its superiority of high sensitivity, high selectivity, and high mass accuracy in structural identification [19,20]. Recently, UHPLC-Q-Exactive Plus Orbitrap MS has been used for improving the efficiency of metabolite identification. This technology would be a powerful tool for the characterization and identification of metabolites even at low concentrations in vivo and in vitro [21]. In this study, UHPLC-Q-Exactive Plus Orbitrap MS combined with network pharmacology was used to systematically study the metabolic characteristics of neobavaisoflavone in vivo and in vitro for the first time.

In the present study, the SPE method, methanol precipitation, and acetonitrile precipitation were used to prepare biological samples. The samples were analyzed on UHPLC-Q-Exactive Plus Orbitrap MS. The spectra were analyzed by Thermo Xcalibur 3.0 workstation. Based on parallel reaction monitoring (PRM), diagnostic product ions (DPIs), and neutral loss fragments (NLFs), neobavaisoflavone and its metabolites were preliminarily identified in plasma, urine, feces, the liver, and the liver microsome of rats. The possible metabolic pathways of neobavaisoflavone were mapped according to metabolic reactions and metabolites. The Swiss ADME and the Swiss Target Prediction databases were used to predict active metabolites and targets, and Cytoscape version 3.8.2 was used to draw an ingredient-target network. The GO and KEGG analysis of neobavaisoflavone and its metabolites were performed on the Metascape database. Our findings elucidated the metabolic properties of neobavaisoflavone in vivo and in vitro for the first time, which might also help to explore potential active metabolites and mechanisms. It is expected to be a useful resource for drug development using natural products.

## 2. Results

### 2.1. DPIs Construction and Fragmentation Pathways of Neobavaisoflavone

**Figure 1:**
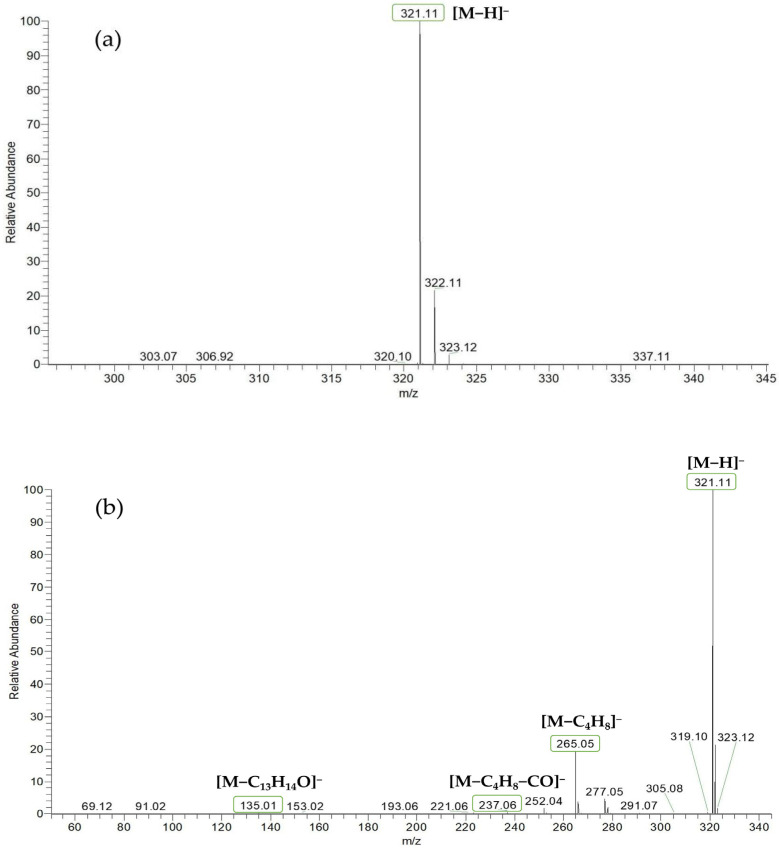
The MS (**a**), and MS^2^ (**b**) spectra of neobavaisoflavone in the negative ion mode.

**Figure 2:**
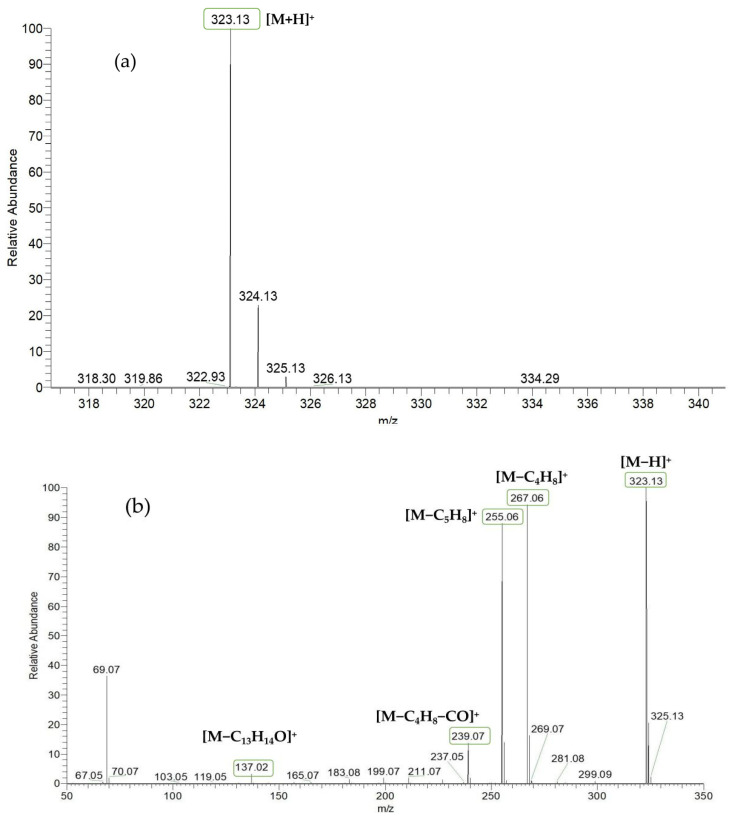
The MS (**a**), and MS^2^ (**b**) spectra of neobavaisoflavone in the positive ion mode.

**Figure 3:**
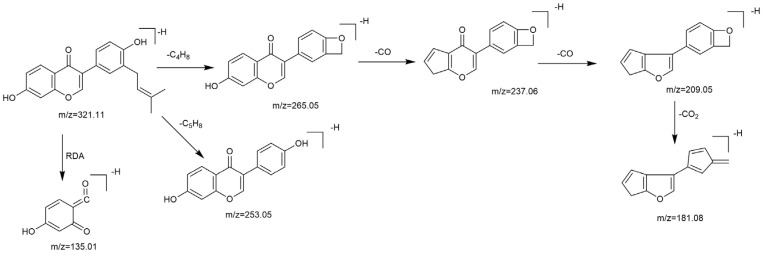
The proposed fragmentation pathways for neobavaisoflavone in the negative ion mode.

**Figure 4:**
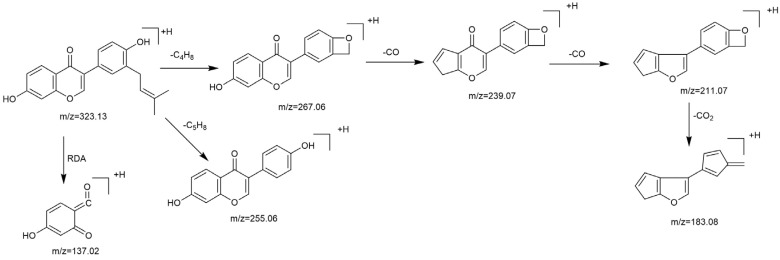
The proposed fragmentation pathways for neobavaisoflavone in the positive ion mode.

Based on the mass spectral fragmentation pathways reported in the literature and the fragment information of neobavaisoflavone, the result showed that neobavaisoflavone generated the [M−H]^−^ ion at *m/z* 321.11 (C_20_H_17_O_4_, t_R_ 11.47 min, 0.316 ppm) and the [M+H]^+^ ion at *m/z* 323.13 (C_20_H_19_O_4_, t_R_ 11.47 min, 3.700 ppm). In negative ion mode, characteristic fragment ions were detected: *m/z* 265.05 [M−H−C_4_H_8_]^−^, *m/z* 253.05 [M−H−C_5_H_8_]^−^, *m/z* 237.06 [M−H−C_4_H_8_−CO]^−^, and *m/z* 209.05 [M−H−C_4_H_8_−CO−CO]^−^. The fragment ion at *m/z* 135.01 was formed by retro-Diels-Alder (RDA) fission. In positive ion mode, characteristic fragment ions were detected: *m/z* 267.06 [M+H−C_4_H_8_]^+^, *m/z* 255.06 [M+H−C_5_H_8_]^+^, *m/z* 239.07 [M+H−C_4_H_8_−CO]^+^, *m/z* 211.07 [M+H−C_4_H_8_−CO−CO]^+^, and *m/z* 183.08 [M+H−C_4_H_8_−CO−CO−CO_2_]^+^. The fragment ion at *m/z* 137.02 was formed by RDA fission. The DPIs and NLFs of neobavaisoflavone provided guidance for the subsequent identification of metabolites. The MS (a), and MS^2^ (b) spectra and proposed fragmentation pathways of neobavaisoflavone were summarized, as shown in Table 1 and Figure 1, Figure 2, Figure 3 and Figure 4.

### 2.2. Fragmentation Pattern Analysis and DPIs Determination of Neobavaisoflavone Metabolites

UHPLC-Q-Exactive Plus Orbitrap MS was used to analyze the biological samples of urine, plasma and feces, and liver and liver microsome. The spectra were analyzed by Thermo Xcalibur 3.0 workstation. Based on PRM, DPIs, and NLFs, a total of 72 metabolites were preliminarily identified. The chromatographic and mass spectrometric information from all the metabolites was summarized in Table 2.

Metabolite M45 was eluted at 8.16 min with [M−H]^−^ ion at *m/z* 307.10 (C_19_H_15_O_4_, 0.536 ppm). In ESI-MS^2^ spectra, the fragment ions at *m/z* 279.07, 266.06, 253.05 were produced by the losses of C_2_H_4_, C_3_H_5_, and C_4_H_6_, respectively. The result showed M45 and its isomers (M7, M55) may be the compounds formed by demethylation of neobavaisoflavone.

M66, with [M−H]^−^ ion at *m/z* 335.09, was eluted at 11.15 min (C_20_H_15_O_5_, 0.179 ppm). In negative ion mode, the characteristic fragment ions at *m/z* 317.21 and 299.20 were generated by the successive loss of H_2_O. The fragment ion at *m/z* 291.23 was another characteristic ion. M66 was tentatively identified as the oxidation product of neobavaisoflavone, which was formed by the oxidation of methylene to ketone.

M67 presented a [M−H]^−^ ion at *m/z* 319.10 (C_20_H_15_O_4_, 0.140 ppm), and was eluted at 12.67 min. In the negative ESI-MS/MS spectra, the ion at *m/z* 304.07 was formed by the loss of CH_3_. The ion at *m/z* 116.93 was formed by the consecutive losses of C_9_H_5_O_3_ and C_3_H_6_. Therefore, M67 was tentatively identified as a cyclization product of neobavaisoflavone.

M29 was eluted at 7.21 min and had [M−H]^−^ ion at *m/z* 335.13 (C_21_H_19_O_4_, error with −4.314 ppm). The fragment ion at *m/z* 265.05 was formed due to the losses of CH_3_ and C_4_H_7_. The ion at *m/z* 321.11 was another characteristic fragment ion. These results indicated that M29 was the product of methylated neobavaisoflavone.

M39, M48, and M61 were separated by elution at 7.96, 8.22, and 9.45 min, respectively, with errors −0.325 ppm, 0.206 ppm, and −1.151 ppm, and had [M−H]^−^ ion at *m/z* 339.12. It was demonstrated that the molecular formula was C_20_H_19_O_5_ by the mass weight measurement. In the MS/MS spectra, characteristic fragment ions of *m/z* 267.06, *m/z* 252.04, and *m/z* 237.05 were generated by the successive losses of C_4_H_9_ and CH_3_. Therefore, M39, M48, and M61 were recognized as hydrolyzed products of neobavaisoflavone.

M37, eluted at 7.90 min, had [M−H]^−^ ion at *m/z* 337.11 (C_20_H_17_O_5_, error with 0.652 ppm), which was 16 Da (O) more than that of neobavaisoflavone. In the ESI-MS/MS spectra, the fragment ion at *m/z* 319.10 was generated by the loss of H_2_O. The fragment ions at *m/z* 265.05 and *m/z* 252.04 were formed by the losses of C_4_H_8_O and C_5_H_9_O, respectively. Metabolites M41, M42, M54, M63, and M64 had the same fragment ions as M37. They were separated by elution at 8.06 min, 8.13 min, 8.75 min, 9.73 min, and 10.40 min with errors as 1.008 ppm, 1.097 ppm, 1.097 ppm, 0.445 ppm, −0.060 ppm, respectively. It was speculated that they might be the compounds produced by the oxidation reaction of neobavaisoflavone.

M43 was separated by elution at 8.15 min with [M−H]^−^ ion at *m/z* 351.09 (C_20_H_15_O_6_, 0.841 ppm). The main fragment ions were *m/z* 307.10 and 252.04. They were obtained by the losses of C_2_H_4_O and C_5_H_7_O_2_, respectively. These results showed that M43 might be a carbonylation product of M37.

Metabolite M62 was eluted at 9.65 min with [M−H]^−^ at *m/z* 351.12 (C_21_H_19_O_5_, 0.140 ppm). In the negative ESI-MS/MS spectra, the ion at *m/z* 321.11 was fragmented as [M-H-OCH_2_]^−^. The fragment ions at *m/z* 307.19 and 271.21 were formed by the losses of CH_2_ and two H_2_O. It was speculated that this metabolite was produced by the methylation reaction after the hydroxylation of neobavaisoflavone.

M8, with a retention time of 5.83 min, had [M−H]^−^ ion at *m/z* 369.10 (C_20_H_17_O_7_, −4.252 ppm), which was 48 Da less than the mass of M0. The main fragment ions in the ESI-MS/MS spectra were at *m/z* 193.05, 175.02, and 124.01. Thus, M8 was identified as a triple hydroxylation product of M0.

Neobavaisoflavone underwent a reduction reaction to generate a series of products, among which M44, and M50 had [M−H]^−^ ion at *m/z* 307.13 (C_20_H_19_O_3_), with retention time at 6.10 min, 8.46 min, and errors as −1.273 ppm, −4.855 ppm, respectively. In ESI-MS^2^ spectra, the fragment ions at *m/z* 236.10, 161.10, 252.01 were formed as [M−H−OH−C_4_H_6_]^−^, [M−H−C_9_H_6_O_2_]^−^ and [M−H−C_4_H_7_]^−^, respectively. M19, M69, and M70 had [M−H]^−^ ion at *m/z* 295.13 (C_19_H_19_O_3_), with retention time at 8.68 min, 17.35 min, 19.06 min and errors as −4.544 ppm, −3.188 ppm, −4.950 ppm, respectively. In ESI-MS^2^ spectra, the fragment ions at *m/z* 277.11, 135.08, 121.06 were formed as [M−H−H_2_O]^−^, [M−H−C_11_H_12_O]^−^ and [M−H−C_11_H_10_O_2_]^−^, respectively.

The [M−H]^−^ of M71 was at *m/z* 482.13 and the retention time was 23.06 min. It was speculated that its most probable molecular formula was C_25_H_24_O_7_NS with an error of −2.591 ppm. The ion at *m/z* 129.97 was the neutral fragment of N-acetylcysteine. The fragment ion at *m/z* 116.93 was obtained by the loss of CH_3_ from N-acetylcysteine. The fragment ion at *m/z* 412.21 was a characteristic ion as well. It was speculated that M71 was a metabolite produced by the S-binding of N-acetylcysteine in neobavaisoflavone.

M13 was eluted at 6.00 min with [M−H]^−^ at *m/z* 497.14 (C_26_H_25_O_10_, error with 0.617 ppm). In the ESI-MS/MS spectra, the characteristic ion at *m/z* 321.11 was generated by the loss of GluA. The fragment ion at *m/z* 265.02 was produced by the loss of C_4_H_8_. The ions at *m/z* 175.02 and 113.02 were the characteristic fragment ions of GluA in negative ion mode. M47, M51, and M52 were detected at 8.21 min, 8.52 min, and 8.66 min (errors with 0.375 ppm, 0.436 ppm, 0.255 ppm) with the same fragment ions as M13. It was speculated that they might be the metabolites produced by the glucuronidation of neobavaisoflavone.

M56, M58, and M60 were separated by elution at 9.08, 9.18, and 9.33 min, and yielded [M−H]^−^ ion at *m/z* 401.07 (C_20_H_17_O_7_S, errors with −0.249 ppm, 0.972 ppm, −0.175 ppm). In the ESI-MS/MS spectra, the fragment ion at *m/z* 321.11 was generated by the loss of SO_3_ (*m/z* 79.96). The fragment ion at *m/z* 121.03 was formed by the loss of O after RDA fission. These metabolites were 80 Da higher than that of M0. It was speculated that they might be the metabolites produced by the sulfation of neobavaisoflavone.

M12, M33, M34, and M40 were eluted at 5.99 min, 7.56 min, 7.74 min, and 7.97 min, and had [M−H]^−^ ion at *m/z* 673.18 (C_32_H_33_O_16_, error with 0.667 ppm, 0.028 ppm, 0.578 ppm, 0.845 ppm). In the ESI-MS/MS spectra, the characteristic fragment ions at *m/z* 497.14, 337.11 were generated by the successive losses of two GluA. The ions at *m/z* 175.02 and 113.02 were the characteristic fragment ions of GluA in the negative ion mode. It was speculated that they might be the metabolites produced by the glucuronidation of M13.

M4, M6, M9 M11, M16, M18, and M20 were eluted at 5.23 min, 5.78 min, 5.84 min, 5.97 min, 6.21 min, 6.37 min, and 6.45 min, with [M−H]^−^ ion at *m/z* 513.14 (C_26_H_25_O_11_, error with 3.258 ppm, −1.029 ppm, −0.074 ppm, 0.160 ppm, −0.074 ppm, −0.561 ppm, −0.561 ppm). In the ESI-MS/MS spectra, the fragment ions at *m/z* 337.11, and 319.11 were generated by the successive loss of GluA and H_2_O. The ions at *m/z* 175.02 and 113.02 were the characteristic fragment ions of GluA in the negative ion mode. It was speculated that they might be the metabolites produced by the hydroxylation or cyclization reaction of M13.

M57 generated [M−H]^−^ ion at *m/z* 469.15 (C_25_H_25_O_9_, 4.138 ppm), which was 28 Da less than M13. The ESI-MS^2^ spectra contained *m/z* 401.07, *m/z* 293.17, and *m/z* 275.16, which were generated by the successive losses of C_5_H_8_ or GluA and O. The fragment ions at *m/z* 175.02 and 113.02 were the characteristic fragment ions of GluA. These results indicated that M57 was the product of the decarbonylation of M13.

M59 gave rise to a [M−H]^−^ ion at *m/z* 511.16 (C_27_H_27_O_10_, 0.072 ppm). It was 14 Da larger than M13, revealing that methylation reaction might have occurred in vivo. In its ESI-MS/MS spectrum, a train of fragment ions at *m/z* 320.10 ([M−H−GluA−CH_3_]^−^), *m/z* 265.05 ([M−H−GluA−C_5_H_10_]^−^) supported our initial conjecture. In addition, the ions at *m/z* 175.02 and 113.02 were the characteristic fragment ions of GluA. Therefore, M59 was ultimately identified as the methylation product of M13.

M14 gave rise to a [M−H]^−^ ion at *m/z* 515.15 (C_26_H_27_O_11_, −0.132 ppm). It was 18 Da greater than M13. In its ESI-MS/MS spectrum, there was a train of fragment ions at *m/z* 339.12 ([M−H−GluA]^−^), *m/z* 323.13 ([M−H−GluA−O]^−^), and *m/z* 266.05 ([M−H−GluA−C_4_H_9_O]^−^). In addition, the ions at *m/z* 175.02 and 113.02 were the characteristic fragment ions of GluA. Hence, M14 was ultimately identified as a hydration product of M13.

M15, M17, and M48 with the same [M−H]^−^ ion at *m/z* 527.16 (C_27_H_27_O_11_, errors with −1.514 ppm, −3.942 ppm, −0.470 ppm) were detected at 5.93, 6.23 and 8.21 min. It was 30 Da larger than M13. In the negative ion mode, there were fragment ions at *m/z* 351.12 ([M−H−GluA]^−^), *m/z* 336.10 ([M−H−GluA−CH_3_]^−^), and *m/z* 280.04 ([M−H−GluA−C_5_H_11_]^−^). Furthermore, the ions at *m/z* 175.02 and 113.02 were the characteristic fragment ions of GluA. It was speculated that they might be the metabolites produced by the hydroxymethylation reaction of M13.

M31 and M32 yielded [M−H]^−^ at *m/z* 577.10 in the negative ion mode, and the retention times were 7.28 min and 7.36 min, respectively. It was speculated that the molecular formula was C_26_H_25_O_13_S, and the errors were 0.281 ppm and −0.048 ppm, respectively. The fragment ion at *m/z* 497.14 was obtained by the loss of the SO_3_ (*m/z* 79.96), the fragment ion at *m/z* 401.07 was obtained by the loss of GluA, and the fragment ion at *m/z* 321.11 was formed by the successive losses of GluA and SO_3_. M31 and M32 might be the sulfation and glucuronidation products of M0. Metabolite M5 had [M−H]^−^ ion at *m/z* 593.10 (C_26_H_25_O_14_S, −0.105 ppm) with a retention time of 5.37 min. It was 16 Da greater than M31 and M32. In ESI-MS^2^ spectra, there were characteristic fragment ions of GluA and SO_3_, such as ions at *m/z* 79.96, 175.02, and 113.02. M5 was the metabolite produced by the sulfation, glucuronidation, and hydroxylation of M0.

Many metabolites were produced by a series of reduction reactions of neobavaisoflavone. In the negative ion mode, M1 (C_20_H_19_O_7_S, [M−H]^−^ *m/z* 403.12, error as 4.689 ppm) was eluted at 2.26 min, with fragment ions at *m/z* 227.09, 212.07 obtained by the successive losses of C_9_H_6_O_3_, CH_2,_ and CH_3_. The characteristic fragment ion at *m/z* 113.02 was generated from the successive losses of C_7_H_6_O_3_, C_4_H_8,_ and SO_4_H. The ion at *m/z* 96.96 was SO_4_H. M1 might be the metabolite produced by the reduction of M56. Metabolite M2 (C_20_H_21_O_6_S, [M−H]^−^ *m/z* 389.11, error as 2.684 ppm), eluted at 2.31 min, generated characteristic fragment ions at *m/z* 309.07 and 242.01 by the loss of SO_3_ and C_9_H_7_O_2_, respectively. M2 might be the metabolite produced by the reduction reaction of M56. M3 (C_20_H_19_O_5_S, [M−H]^−^ *m/z* 371.10, error as 1.102 ppm) generated characteristic fragment ions at *m/z* 113.02 and 133.06. M3 might be conjectured as the metabolite produced by the reduction reaction of M56. M35 was speculated as the metabolite produced by the carbonyl reduction of M56 with a retention time of 6.46 min and yielded [M-H]^−^ ion at *m/z* 387.09 (C_20_H_19_O_6_S, 0.658 ppm). In its ESI-MS^2^ spectra, there were characteristic fragment ions at *m/z* 371.24, 319.14, 143.11, and 113.02.

M10, M22–28, M36, and M46 possessed the same [M−H]^−^ ion at *m/z* 417.06 (C20H17O8S). In the ESI-MS/MS spectra, the characteristic fragment ions at *m/z* 337.11, and 321.11 were generated by the losses of SO_3_ (*m/z* 79.96) and SO_4_. The ion at *m/z* 307.10 was [M-H-SO_4_-CH_2_]^−^. Because the neobavaisoflavone had a benzene ring and hydroxyl, many isomeric products were generated when the sulfation reaction and the hydroxylation reaction occurred. It was speculated that they might be the metabolites produced by the hydroxylation reaction of M56.

M38 yielded [M−H]^−^ ion at *m/z* 415.09 (C_21_H_19_O_7_S, with error as 1.012 ppm) with a retention time of 7.90 min, which was 14 Da more massive than M56. The ions at *m/z* 335.09 ([M−H−SO_3_]^−^), and 145.05([M−H−SO_3_−C_10_H_8_O_3_−CH_2_]^−^) were generated in their ESI-MS/MS spectra. The ion at *m/z* 151.02 was generated by the RDA fission. The ion at *m/z* 131.03 was produced from RDA fission and the successive losses of SO_3_ and C_4_H_7_. Therefore, metabolite M38 was tentatively identified as the methylation product of M56.

M21 and M30 presented a [M−H]^−^ ion at *m/z* 433.06 with retention times at 6.51 min and 7.26 min, respectively. It was speculated that the molecular formula was C_20_H_17_O_9_S, and the errors were −1.268 ppm and −2.192 ppm, respectively. The fragment ions at *m/z* 418.13 and 403.09 were obtained by the successive losses of the two CH_3_. The ion at *m/z* 96.96 was SO_4_H. The fragment ions at *m/z* 281.04 and 218.06 were obtained by the loss of OH or SO_3_ after RDA fission. These metabolites were 32 Da more massive than M56, and they might be inferred as double hydroxylation products of M56. Metabolite M68, with [M−H]^−^ ion at *m/z* 449.06 (C_20_H_17_O_10_S, −0.632 ppm), had a retention time of 15.44 min. In ESI-MS^2^ spectra, there were characteristic fragment ions at *m/z* 379.21, 151.02, 298.05, and 96.96. M68 was 48 Da more massive than M56. So, it could be speculated that M68 was the metabolite produced by the triple hydroxylation reaction of M56.

The [M−H]^−^ ion of M72 was at *m/z* 481.03 and the retention time was 23.09 min. It was speculated that its molecular formula was C_20_H_17_O_10_S_2_, and the error was 3.546 ppm. The fragment ion at *m/z* 400.74 was obtained by the neutral loss of SO_3_. The neutral fragment ion SO_4_H was at *m/z* 96.96, with the DPIs at *m/z* 412.21 [M−C_5_H_9_]^−^ and *m/z* 129.97 [M−H−2SO_3_−2O−C_9_H_4_O_2_−CH_3_]^−^, it could be speculated that M72 might be a metabolite produced by the sulfation of M56. The [M−H]^−^ ion of M53 and M65 was at *m/z* 497.02 with the retention times at 8.66 min and 10.61 min, respectively. It was speculated that the molecular formula was C_20_H_17_O_11_S_2_, and the errors were 2.054 ppm and 3.040 ppm, respectively. The neutral losses of SO_3_, SO_3,_ and O might obtain fragment ions at *m/z* 321.11. The ion at *m/z* 113.02 was [M−H−2SO_3_−2OH−C_9_H_4_O_3_−2CH_3_]^−^. Therefore, it was speculated that the metabolites M53 and M65 might be produced by the hydroxylation and sulfation of M56.

### 2.3. Metabolic Pathway Mechanism Analysis of Neobavaisoflavone

**Figure 5:**
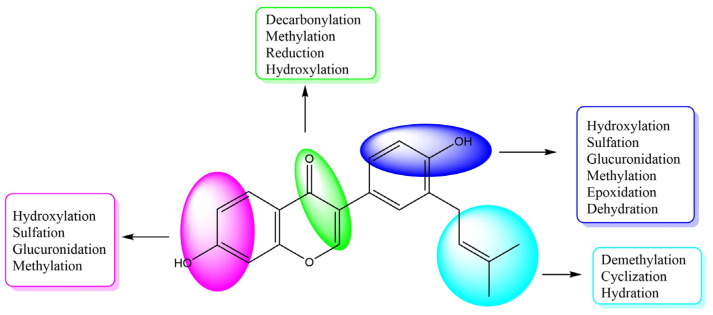
The summarized metabolic features of neobavaisoflavone in rats.

**Figure 6:**
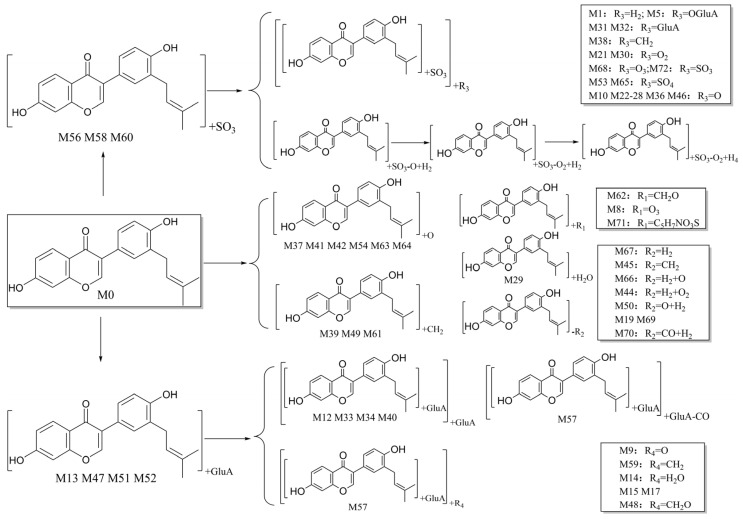
The proposed neobavaisoflavone metabolic pathways in vivo and in vitro.

After the neobavaisoflavone was absorbed, 28 metabolites were detected in plasma samples, 43 in urine samples, 18 in feces samples, and six in the liver samples. In addition, four metabolites were detected in liver microsome samples. Neobavaisoflavone was detected in all samples except for liver samples. These data showed that it would be metabolized and excreted in urine and feces partially after neobavaisoflavone was absorbed into the blood through the gastrointestinal tract. The products of glucuronidation, sulfation, oxidation and cyclization were identified in plasma samples. In the urine samples, metabolites were formed by phase I metabolism, such as hydration, dehydration, oxidation, and reduction reactions. Hydroxylated products were found primarily in feces samples. There were few metabolites in liver microsome, and they were only sulfated products and methylated products in vitro. The metabolic features of neobavaisoflavone in rats are shown in Figure 5. The metabolic pathway of neobavaisoflavone is shown in Figure 6.

### 2.4. Network Pharmacology of Neobavaisoflavone and Its Main Metabolites

From the above metabolic pathways, it is speculated that neobavaisoflavone might be one of the main components that exerted its efficacy. The Swiss ADME database (http://www.swissadme.ch/, accessed on 26 February 2022) and the Swiss Target Prediction database (http://www.swisstargetprediction.ch/, accessed on 26 February 2022) were used to screen the active metabolites and their targets. Neobavaisoflavone and its eight metabolites had high bioavailability scores and drug-likeness (Table 3). These eight components were products of sulfation, hydroxylation (including isomers), demethylation, hydration, epoxidation, decarbonylation reduction, and methylation. The bioavailability of metabolites produced by phase II metabolism was reduced. Hence, it was speculated that the components with drug-likeness might be neobavaisoflavone, sulfated metabolites, and other metabolites formed by phase I metabolism. Finally, nine components were used for further research in network pharmacology via the Metascape database [22] (https://metascape.org/, accessed on 26 February 2022).

#### 2.4.1. Construction and Analysis of Ingredient-Target Network

**Figure 7:**
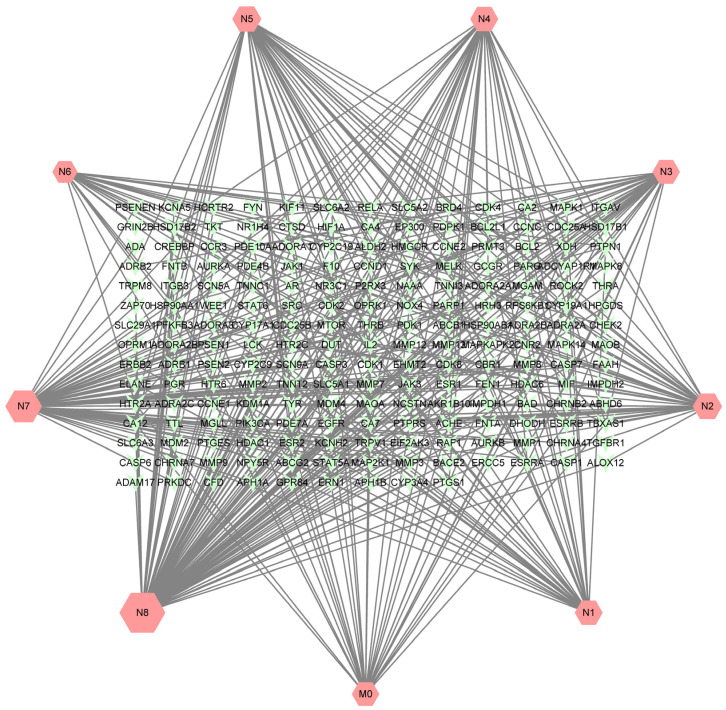
The ingredient-target network of neobavaisoflavone and its main metabolites, and the size was correlated to the degree of targets.

**Figure 8:**
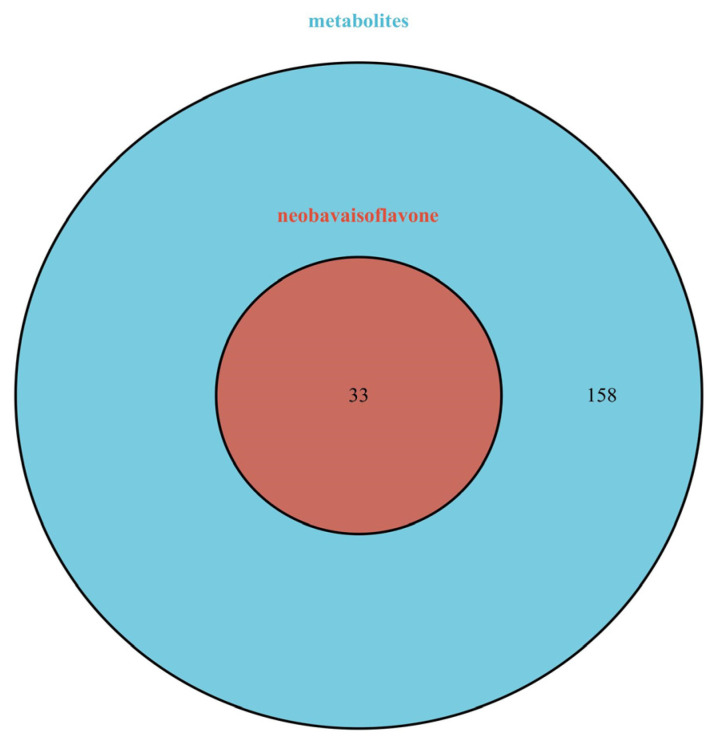
The Venn diagram of targets between neobavaisoflavone and its metabolites. Blue: targets of metabolites; red: targets of neobavaisoflavone.

A total of 405 targets were obtained by Swiss Target Prediction, and 191 gene targets remained after removing deduplicates. On the basis of these data, an ingredient-target network was constructed through Cytoscape version 3.8.2. As shown in Figure 7, the network consisted of 200 nodes and 405 edges. There were 33 targets of neobavaisoflavone, while the metabolite (N8) had 99 targets, and the metabolite (N7) had 66 targets. It was indicated that potential pharmacological effect changed, and metabolites of neobavaisoflavone might play a larger role. A Venn diagram was drawn to distinguish neobavaisoflavone from its metabolites, as shown in Figure 8. The targets of neobavaisoflavone were all included in the targets of the metabolites, which further verified that the metabolites had a more potent possibility. It was speculated that N7 and N8 were the main components that exerted pharmacological effects among the above 9 components.

#### 2.4.2. GO Analysis of Target

**Figure 9:**
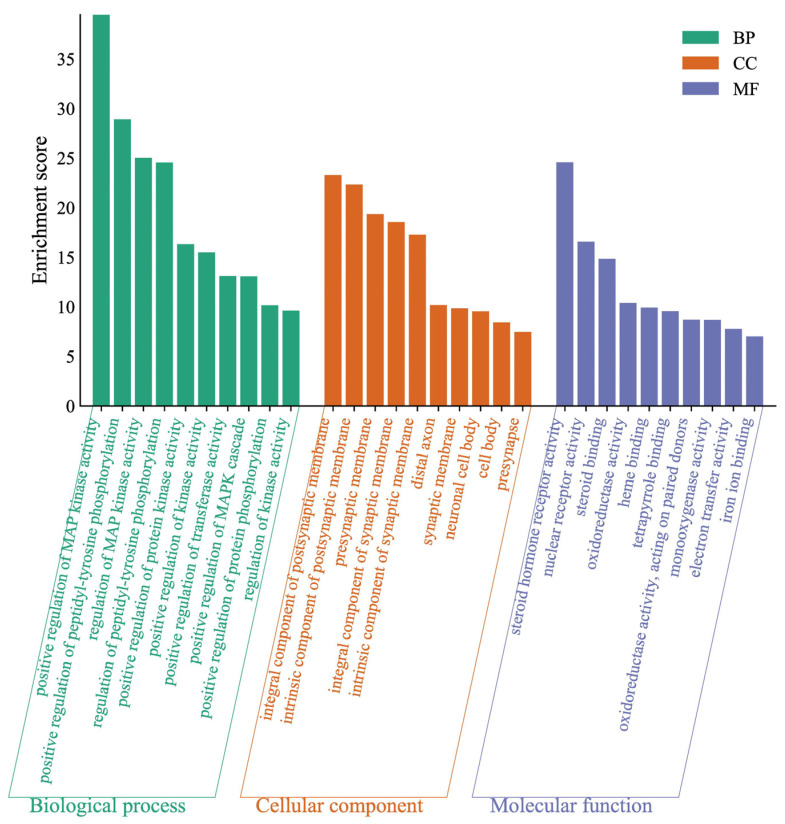
Gene Ontology (GO) enrichment analysis of the target proteins of neobavaisoflavone.

The difference in gene targets might result in pharmacological changes. The enriched terms in BP, MF, and CC categories were selected according to *p* values less than 0.01. To neobavaisoflavone (Figure 9), the target proteins were mainly involved in the positive regulation of MAP kinase activity, positive regulation of peptidyl-tyrosine phosphorylation, and regulation of MAP kinase activity in the BP category; the target proteins were classified into steroid hormone receptor activity, nuclear receptor activity and steroid binding in the MF category; the target proteins were mainly involved in integral component of postsynaptic membrane, an intrinsic component of the postsynaptic membrane and presynaptic membrane in the CC category.

**Figure 10:**
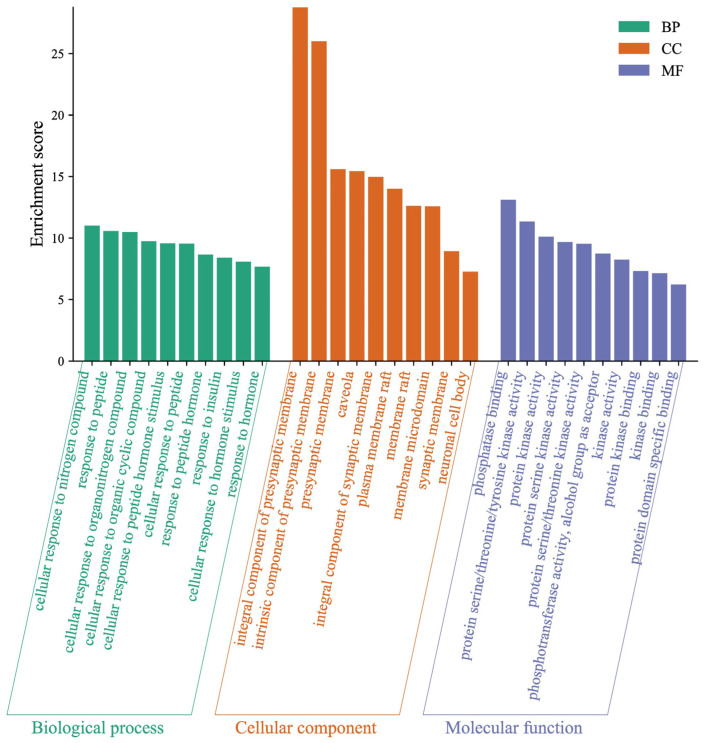
Gene Ontology (GO) enrichment analysis of the target proteins of neobavaisoflavone combined with its metabolites.

To neobavaisoflavone metabolites (Figure 10), the target proteins were mainly involved in the cellular response to nitrogen compound, response to peptide, and cellular response to organonitrogen compound in the BP category; the target proteins were classified into phosphatase binding, protein serine/threonine/tyrosine kinase activity and protein kinase activity in the MF category; the target proteins were classified into an integral component of the presynaptic membrane, an intrinsic component of the presynaptic membrane and presynaptic membrane in the CC category. The GO enrichment analysis results verified that the metabolites expanded the functional areas and types compared to neobavaisoflavone.

#### 2.4.3. KEGG Analysis of Target

**Figure 11:**
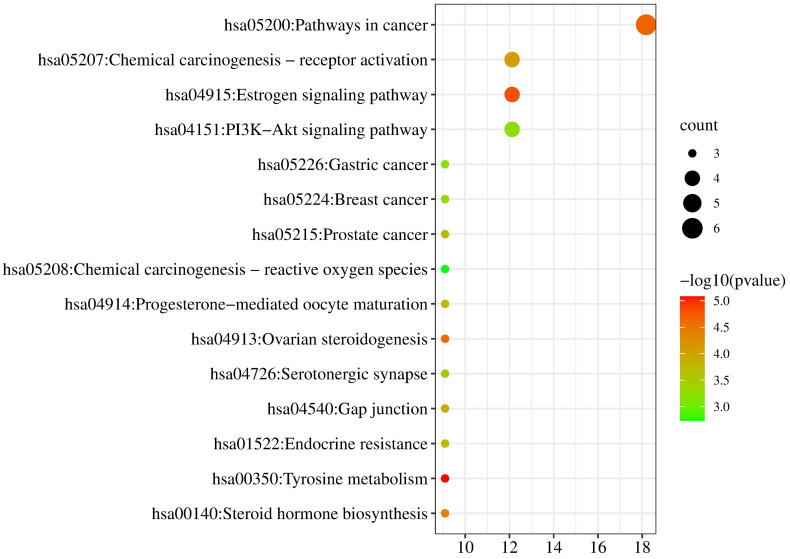
KEGG analysis of the target proteins of neobavaisoflavone.

**Figure 12:**
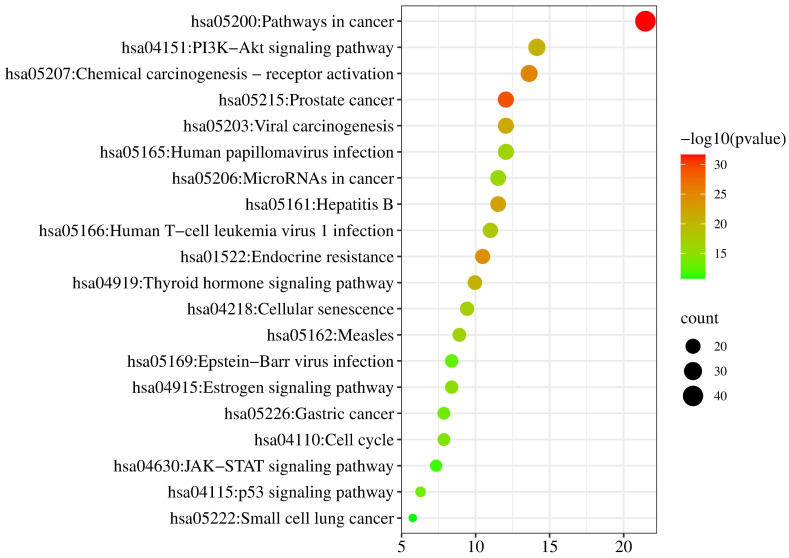
KEGG analysis of the target proteins of neobavaisoflavone and its metabolites.

KEGG pathways were enriched from the Metascape database (*p* < 0.01). Then the relationships between pathways and pharmacological effect mechanism were predicted from the KEGG PATHWAY database (https://www.kegg.jp/kegg/pathway.html, accessed on 28 February 2022). The pathways of neobavaisoflavone were mainly involved in Tyrosine metabolism, Estrogen signaling pathway, Ovarian steroidogenesis, Gap junction, Prostate cancer, Serotonergic synapse, and Chemical carcinogenesis-reactive oxygen species (Figure 11). The descriptions of these pathways from the KEGG PATHWAY database could be corroborated in the literature about neobavaisoflavone. For the neobavaisoflavone metabolites, the number of pathways increased significantly. The pathways mainly involved were Pathways in Cancer, PI3K-Akt signaling pathway, Chemical carcinogenesis-receptor activation, Endocrine resistance, Neuroactive ligand-receptor interaction, Lipid and atherosclerosis, cAMP signaling pathway, Progesterone-mediated oocyte maturation, Serotonergic synapse, Notch signaling pathway, and so on (Figure 12). These confirmed that the neobavaisoflavone had anti-inflammatory and anti-cancer effects. In addition, it could be speculated that neobavaisoflavone had potential antioxidant, whitening, anti-osteoporosis, and estrogen-like effects through querying the KEGG database and the literature [9,10,12]. However, the Steroid hormone biosynthesis pathway is related to lipid metabolism. This finding showed that neobavaisoflavone might interfere with lipid metabolism to produce hepatotoxicity after being absorbed into the blood. Meanwhile, the result was mutually confirmed by the published literature [14]. This discovery will provide a new idea for the study of the toxicity mechanism of neobavaisoflavone. To sum up, the KEGG analysis results further verified that the metabolites research provided a new way to study the pharmacological and toxicological mechanisms of neobavaisoflavone.

## 3. Discussion

In this study, a total of 72 metabolites of neobavaisoflavone were initially identified, including 28 in plasma samples, 43 in urine, 18 in feces, six in the liver, and four in liver microsome. Neobavaisoflavone underwent glucuronidation, sulfation, hydroxylation, methylation, cyclization, hydration, and other reactions, then these metabolites were transported to various organs to exert pharmacological effects.

The neobavaisoflavone and eight active metabolites with drug-likeness properties, high bioavailability, and possible pharmacological effects were predicted and screened to perform GO and KEGG analysis. Then their mechanisms of action were predicted by analyzing pathways. For the pathway of tyrosine metabolism, the synthesis of tyrosinase structural analogs that competed with tyrosine might effectively inhibit the production of melanin [12]. This mechanism of neobavaisoflavone might be applied to research whitening cosmetics. Tyrosine might promote the formation of melanin and relieve the symptoms of vitiligo [23], which is a traditional pharmacological effect of neobavaisoflavone. Amino acids associated with this pathway might act as nutritional supplements, then neobavaisoflavone might enhance immunity. Therefore, it was speculated that neobavaisoflavone might play the role of whitening, treating vitiligo, and improving immunity by regulating this pathway. The estrogen signaling pathway was related to many physiological processes of mammals, including reproduction, cardiovascular protection, and bone integrity [24]. The other pathways were related to anticancer and estrogen-like effects [11,25,26]. Besides the pharmacological mechanisms of anti-osteoporosis, anti-cancer, and estrogen-like effects, the pathways were also related to anti-inflammatory, antioxidant, neuroactive, and adrenergic effects [27,28]. In addition, the results of KEGG analysis also showed that neobavaisoflavone might interfere with lipid metabolism to produce toxicity through the steroid hormone biosynthesis pathway, which was consistent with the literature [14] that the neobavaisoflavone had certain hepatotoxicity. In a word, it was indicated that the metabolites provided more possibilities compared with neobavaisoflavone in pharmacological effects.

## 4. Materials and Methods

### 4.1. Reagents and Chemicals

The Neobavaisoflavone reference (LOT: MUST-21072205, Purity: 99.62%) was purchased from Chengdu Must Biotechnology Co., Ltd. (Chengdu, China). Rat liver microsome (LOT: 20210305) were purchased from Wuxi Xinrun Biotechnology Co., Ltd. (Wuxi, China). Grace Pure TM SPE C_18_-Low solid-phase extraction cartridges (200 mg/3 mL, 59 μm, 70 Å) were obtained from Grace Davison Discovery Science (Deerfield, IL, USA). All the acetonitrile, methanol, and formic acid of HPLC grade were purchased from Thermo Fisher Scientific (Fair Lawn, NJ, USA). Water was purchased from Watsons Food & Beverage Co., Ltd. (Guangzhou, China).

### 4.2. Animals and Drug Administration

Male SD rats weighing 200 ± 10 g were obtained from Jinan Pengyue Laboratory Animal Technology Co., Ltd. and raised at Binzhou Medical University. All the rats were living under stable controlled conditions at standard temperature (24 ± 2 °C) and humidity (50 ± 10%) and kept on a 12 h light/12 h dark regime with free access to food and water. Following 3 days of continuous acclimatization to the environment, the rats were randomly divided into two groups: the control group (n = 3) and the treatment group (n = 3) [29,30]. The treatment group was given neobavaisoflavone reference, which was suspended in normal saline by gavage at a dose of 150 mg/kg, while the control group was administered with an equal amount of normal saline. The rats were administered for 3 days consecutively and were fasted but had free access to water 12 h before the experiment. The study was conducted in accordance with the Institutional Animal Care and Use Committee in Binzhou Medical University (2021-085) recognized principles for the use and care of laboratory animals.

### 4.3. Biological Samples Collection and Preparation

#### 4.3.1. Plasma Samples

At 0.5, 1, 1.5, 2, 4, and 6 h after the last administration, 0.5 mL of blood was collected from the orbital vein of the rats, respectively. The blood samples of the same group were mixed and placed in a centrifuge tube coated with heparin sodium, and they were centrifuged at 3500 r/min for 10 min (4 °C). The supernatant was obtained and frozen at −80 °C. There were three methods for precipitation and concentration of protein, such as the SPE method, methanol precipitation, and acetonitrile precipitation. Firstly, the SPE column was activated. Then 1 mL of plasma sample was loaded. It was washed with 3 mL of water and eluted with 3 mL of methanol. Then, the methanol eluate was collected in a 5 mL EP tube. Secondly, 3 mL methanol was added to 1 mL plasma for protein precipitation, and the supernatant was collected after centrifugation at 12,000 rpm. Thirdly, 3 mL acetonitrile was added to 1 mL plasma for protein precipitation, and the supernatant was collected after centrifugation at 12,000 rpm.

#### 4.3.2. Urine, Feces, and Liver Tissue Samples

The two groups of rats were placed in metabolic cages, then the urine and feces of the rats in each group were collected within 24 h after the last administration. The urine samples were frozen in the refrigerator at −80 °C after being centrifuged at 12,000 rpm for 15 min. The feces samples were freeze-dried and ground into powder. The powder (2 g) was dissolved with water (10 mL), sonicated for 0.5 h, and centrifuged to obtain the supernatant. The liver tissue was quenched in liquid nitrogen and stored at −80 °C. 1 g liver tissue was added with 10 mL of normal saline to grind, and it was centrifuged at 3500 rpm to get the supernatant. The SPE method was used to process these samples.

#### 4.3.3. Liver Microsome

MgCl_2_ and liver microsome were dissolved in PBS (pH = 7.4), formulated into a solution with MgCl_2_ (3 mM) and protein (1 mg/mL). The solution was used to dissolve the neobavaisoflavone reference with a final drug concentration of 0.1 mg/mL. For the treatment group, 900 μL of the above mixture was added to each well of the 6-well plate, and the control group was given an equal volume of normal saline. After preheating at 37 °C for 5 min, 100 μL NADPH (final concentration 25 mg/mL) was added to start the reaction and continued to incubate at 37 °C. Then 100 μL system solution was added with 200 μL of cold acetonitrile to terminate the reaction at 5, 10, 15, 30, 45, 60, 120, and 240 min. Then, the supernatant was obtained after being centrifuged at 12,000 rpm for 15 min.

All of the methanol eluate and supernatant was dried with N_2_ at room temperature, respectively. The residue was redissolved with 300 μL methanol and centrifuged at 12,000 rpm for 15 min.

### 4.4. Instrument and Conditions

Chromatographic analysis was performed on Vanquish UHPLC (Thermo Scientific, Karlsruhe, Germany) with a Waters ACQUITY UPLC BEH C18 column (100 mm × 2.1 mm, 1.7 μm). The flow rate was 0.3 mL/min. The column temperature was 35 °C. The injection volume was 3 μL. For metabolite separation, the mobile phase (A) consisted of acetonitrile, and the mobile phase (B) consisted of 0.1% formic acid-water. The gradient method followed the steps: 0–5.0 min, 95–70% B; 5.0–10.0 min, 70–50% B; 10.0–27.0 min, 50–10% B; 27.0–27.1 min, 10–95% B; 27.1–30.0 min, 95% B.

The Q-Exactive Plus Orbitrap MS (Thermo Scientific, Dreieich, Germany) was equipped with a heated electrospray ionization source (HESI). The high-resolution scanning range was *m/z* 80–1200 at a resolution of 70,000 with AGC target at 3 × 10^6^. The dd-MS^2^ data were obtained at a resolution of 17,500 with AGC target at 1 × 10^6^. The detection modes were positive ion and negative ion. Nitrogen (purity ≥ 99.99%) was used as sheath gas and auxiliary gas, and the flow rate was 45 and 10 (arbitrary units). Capillary temperature was 320 °C, and evaporator temperature was 400 °C. Spray voltage was 3800/3500 V (+/−). Probe heater temperature was 320 °C. The stepped normalized collision energy (NCE) was set at 15, 30, and 45. S-Lens RF Level was 50.00.

### 4.5. Data Processing

A Thermo Xcalibur 3.0 workstation was used for data acquisition and processing. In order to obtain as many ESI-MS/MS fragment ions as possible for the metabolites of neobavaisoflavone, the signal peaks in the positive ion mode with an intensity of not less than 40,000 and the negative ion mode with an intensity of 10,000 were selected for identification. Predictive settings for all parent and fragment ions were based on exact molecular mass, elemental composition, and possible reactions. The parameters were set as follows: C (5–40), H (5–60), O (2–20), S (0–2), N (0–3), and for the number of cyclic unsaturated double bonds (RDB) (3–20), the mass accuracy error was within 10 ppm. In addition, the metabolites were selected by Swiss ADME and the targets were predicted using Swiss Target Prediction. Then the Metascape database was used to perform GO analysis and KEGG analysis, and the Cytoscape of version 3.8.2 was used to draw an ingredient-target network diagram. The visual analysis of GO and KEGG enrichment results was performed using Bioinformatics online platform.

## 5. Conclusions

In this study, different biological samples were pretreated by the SPE method, methanol precipitation, and acetonitrile precipitation for studies in vitro and in vivo. A total of 72 metabolites of neobavaisoflavone were initially identified based on UHPLC-Q-Exactive Plus Orbitrap MS, including 28 in plasma samples, 43 in urine, 18 in feces, six in the liver, and four in liver microsome. A relatively systematic metabolism characterization of neobavaisoflavone was obtained. These results indicated that neobavaisoflavone was mainly excreted in urine after oral administration in rats. Neobavaisoflavone underwent glucuronidation, sulfation, hydroxylation, methylation, cyclization, hydration, and other reactions, then these metabolites were transported to various organs to exert pharmacological effects. The eight active metabolites with high bioavailability and possible pharmacological effects were predicted by network pharmacology. The differences in the pharmacological effects of the neobavaisoflavone and the eight metabolites were compared. These nine components with drug-likeness properties and high bioavailability were screened to perform GO and KEGG analysis. We found that the metabolites provided more possibilities compared with neobavaisoflavone in pharmacological effects. The complex compounds of medicinal plants might serve as a lead for the development of novel drugs. The study on metabolites of natural products would be a useful resource for drug development. In the present study, the metabolic characteristics of neobavaisoflavone in vivo and in vitro were systematically elucidated for the first time. Hence, this would provide the scientific basis for the exploitation of neobavaisoflavone in the food, pharmaceutical, cosmetic and other industries.

## Figures and Tables

**Table 1:** Mass spectral information of neobavaisoflavone in rat plasma, urine, feces, liver, and liver microsome.

No.	t_R_/min	Formula	Theoretical Mass *m/z*	Experimental Mass *m/z*	RDB	Error (ppm)	MS/MS Fragment Ions	PM	PA	PS	U	F	L	LM
[M−H]^−^	11.47	C_20_H_17_O_4_	321.11323	321.11182	12.5	0.316	321.11(100.00),265.05(18.15), 319.10(0.62),237.05(0.44), 135.01(0.40),253.05(0.38)	+	+	+	+	+	−	+
[M+H]^+^	11.45	C_20_H_19_O_4_	323.12779	323.12659	11.5	3.700	323.13(100.00),267.06(95.34), 255.06(87.43),239.07(14.39), 137.02(3.53),	+	+	+	+	+	−	+

Note: t_R_: retention time; PM: methanol-precipitated plasma samples; PA: acetonitrile-precipitated plasma samples; PS: plasma samples prepared by SPE; U: urine samples; F: fecal samples; L: liver samples; LM: liver microsome samples; +: detected; −: undetected.

**Table 2:** Summary of neobavaisoflavone metabolites in rats plasma, urine, feces, liver, and liver microsome.

Peak	t_R_/Min	Formula [M−H]^−^	Theoretical Mass *m/z*	Experimental Mass *m/z*	RDB	Error (ppm)	MS/MS Fragment Ions	Identification Reactions	PM	PA	PS	U	F	L	LM
M1	2.26	C_20_H_19_O_7_S	403.08575	403.08469	11.5	4.689	113.02(100),403.12(99),212.07(65),227.09(41),96.96(31)	M+SO_3_+H_2_	-	-	-	+	-	-	-
M2	2.31	C_20_H_21_O_6_S	389.10590	389.10855	10.5	2.684	389.09(100),113.02(93),149.06(44),213.06(30),242.01(18)	M+SO_3_-O_2_+H_4_	-	-	-	+	-	-	-
M3	2.71	C_20_H_19_O_5_S	371.09595	371.09726	11.5	1.102	371.10(100),113.02(46),133.06(15)	M+SO_3_-O_2_+H_2_	-	+	-	-	-	-	-
M4	5.23	C_26_H_25_O_11_	513.14023	513.14081	14.5	3.258	187.01(100),113.02(2),513.08(1),337.04(1),175.02(1)	M+C_6_H_8_O_7_	-	-	-	+	-	-	-
M5	5.37	C_26_H_25_O_14_S	593.09705	593.09589	14.5	−0.105	187.01(100),79.96(8),337.11(2),113.02(1),417.06(1)	M+SO_3_+C_6_H_8_O_7_	-	-	-	+	-	-	-
M6	5.78	C_26_H_25_O_11_	513.14023	513.13861	14.5	−1.029	337.11(100),113.02(42),513.14(26),175.02(21),319.09(19),265.05(4)	M+C_6_H_8_O_7_	+	+	+	-	-	-	-
M7 *	5.79	C_19_H_15_O_4_	307.09758	307.09665	12.5	4.704	266.05(3.80),278.06(2.09),323.12(1.40),319.10(0.62),	M-CH_2_	-	-	-	-	-	+	-
M8	5.83	C_20_H_17_O_7_	369.09798	369.09531	12.5	−4.252	369.12(100),193.09(57.65),113.02(56),175.02(22),175.07(10),124.01(6)	M+O_3_	-	-	-	+	-	-	-
M9	5.84	C_26_H_25_O_11_	513.13969	513.13910	14.5	−0.074	337.11(100),113.02(56),175.02(26),513.14(19),319.09(9)	M+C_6_H_8_O_6_+O	-	+	-	+	-	-	-
M10	5.93	C_20_H_17_O_8_S	417.06496	417.06415	12.5	0.684	80.96(100),417.07(55),307.18(18),96.96(15),337.11(9)	M+SO_4_	-	-	-	-	+	-	-
M11	5.97	C_26_H_25_O_11_	513.14023	513.13922	14.5	0.160	337.11(100),113.02(87),175.02(45),513.14(14)	M+C_6_H_8_O_7_	-	-	-	+	-	-	-
M12	5.99	C_32_H_33_O_16_	673.17741	673.17676	16.5	0.667	321.11(100),113.02(72),497.14(67),175.02(31),673.18(28),	M+C_12_H_16_O_12_	+	+	-	+	-	-	-
M13	6.00	C_26_H_25_O_10_	497.14532	497.14453	14.5	0.617	321.11(100),113.02(64),497.14(53),175.02(30),252.04(14)	M+C_6_H_8_O_6_	+	+	+	-	-	-	-
M14	6.02	C_26_H_27_O_11_	515.15593	515.15472	13.5	−0.132	339.12(100),113.02(51),175.02(27),266.05(4)	M+C_6_H_8_O_6_+H_2_O	-	-	-	+	-	-	-
M15	5.93	C_27_H_27_O_11_	527.15583	527.15918	14.5	−1.514	113.02(68),175.02(35),351.07(18),527.12(16)	M+C_6_H_8_O_6_+CH_2_O	-	-	-	+	-	-	-
M16	6.21	C_26_H_25_O_11_	513.14023	513.13910	14.5	−0.074	113.02(100),513.14(88),319.10(56),337.11(53),307.10(41),175.02(41)	M+C_6_H_8_O_7_	+	+	+	-	-	-	-
M17	6.23	C_27_H_27_O_11_	527.15583	527.15991	14.5	−3.942	113.02(70),175.02(33),527.12(28),351.05(8)	M+C_6_H_8_O_6_+CH_2_O	-	-	-	+	+	-	-
M18	6.37	C_26_H_25_O_11_	513.14023	513.13885	14.5	−0.561	337.11(100),113.02(78),175.02(43),266.06(30),513.14(26)	M+C_6_H_8_O_7_	-	-	-	+	-	-	-
M19 *	8.68	C_19_H_19_O_3_	295.13397	295.13004	10.5	−4.544	277.11(100),295.12(44),135.08(21),121.06(19)	M-CO+H_2_	-	-	-	+	-	-	-
M20	6.45	C_26_H_25_O_11_	513.14023	513.13885	14.5	−0.561	513.14(100),113.02(79),337.11(73),319.10(39),175.02(31)	M+C_6_H_8_O_7_	+	+	-	-	-	-	-
M21	6.51	C_20_H_17_O_9_S	433.05985	433.05823	12.5	−1.268	418.13(7),403.09(11),96.96(12)	M+SO_3_+O_2_	-	-	-	-	-	+	-
M22	6.56	C_20_H_17_O_8_S	417.06496	417.06390	12.5	0.085	417.06(100),319.10(63),337.11(57),307.11(57),79.96(10)	M+SO_4_	-	-	-	+	-	-	-
M23	6.63	C_20_H_17_O_8_S	417.06496	417.06400	12.5	0.325	417.06(100),337.11(59),319.10(57),307.10(55),79.96(9),96.96(3)	M+SO_4_	+	+	+	+	-	-	-
M24	6.74	C_20_H_17_O_8_S	417.06496	417.06372	12.5	−0.347	417.06(100),319.10(62),337.11(59),307.10(55),79.96(7)	M+SO_4_	-	-	-	+	-	-	-
M25	6.92	C_20_H_17_O_8_S	417.06496	417.06403	12.5	0.397	417.06(100),337.11(65),319.10(59),307.11(52),96.96(17),79.96(8)	M+SO_4_	-	-	-	+	-	-	-
M26	6.99	C20H17O8S	417.06496	417.06418	12.5	0.756	417.06(100),337.11(58),319.10(50),307.10(38)	M+SO4	-	-	-	-	-	+	-
M27	7.11	C_20_H_17_O_8_S	417.06496	417.06403	12.5	0.397	417.06(100),337.11(63),319.10(58),307.11(51),96.96(28),79.96(10)	M+SO_4_	-	-	-	+	+	-	-
M28	7.17	C_20_H_17_O_8_S	417.06496	417.06430	12.5	1.044	337.11(100),417.06(40),319.10(5),307.10(3),96.96(2)	M+SO_4_	+	+	+	+	-	-	-
M29 *	7.21	C_21_H_19_O_4_	335.12888	335.12567	12.5	−4.314	335.13(23),321.11(2),265.05(2)	M+CH_2_	+	+	-	-	-	-	-
M30	7.26	C_20_H_17_O_9_S	433.05985	433.05783	12.5	−2.192	257.08(100),308.10(99),433.11(92),113.02(81),353.10(17),175.02(31)	M+SO_3_+O_2_	-	+	+	+	-	-	-
M31	7.28	C_26_H_25_O_13_S	577.10215	577.10120	14.5	0.281	321.11(100),577.10(79.99),401.07(19.52),79.96(9.21)	M+SO_3_+C_6_H_8_O_6_	+	-	+	-	-	-	-
M32	7.36	C_26_H_25_O_13_S	577.10215	577.10101	14.5	−0.048	321.11(100),577.10(91),401.07(20),79.96(9),113.02(5)	M+SO_3_+C_6_H_8_O_6_	-	-	+	-	-	-	-
M33	7.56	C_32_H_33_O_16_	673.17741	673.17633	16.5	0.028	113.02(100),175.02(63),321.11(34),497.14(24)	M+C_12_H_16_O_12_	-	-	-	+	-	-	-
M34	7.74	C_32_H_33_O_16_	673.17741	673.17670	16.5	0.578	113.02(100),175.02(61),321.11(27),497.14(16)	M+C_12_H_16_O_12_	-	-	-	+	-	-	-
M35	6.46	C_20_H_19_O_6_S	387.09139	387.09244	11.5	0.658	113.02(100),143.11(85),319.14(37),387.16(31),79.96(14)	M+SO_3_-O+H_2_	+	-	-	+	-	-	-
M36	7.88	C_20_H_17_O_8_S	417.06496	417.06430	12.5	1.044	337.11(75),417.06(58),113.02(29),79.96(27),319.10(22), 96.96(19),307.11(15)	M+SO_4_	-	-	-	+	-	-	-
M37 *	7.90	C_20_H_17_O_5_	337.10815	337.10727	12.5	0.652	337.11(100),265.05(9),279.07(6),319.10(3),252.04(3)	M+O	+	-	-	+	+	-	-
M38	7.90	C_21_H_19_O_7_S	415.08605	415.08502	12.5	1.012	335.09(70),145.05(53),415.09(47),131.03(46),151.02(29)	M+SO_3_+CH_2_	-	-	-	-	-	+	-
M39 *	7.96	C_20_H_19_O_5_	339.12380	339.12259	11.5	−0.325	167.11(19),339.12(11),113.06(8),73.03(8)	M+H_2_O	-	-	-	-	+	-	-
M40	7.97	C_32_H_33_O_16_	673.17741	673.17688	16.5	0.845	673.18(100),351.06(72),113.02(43),674.18(34),321.11(32)	M+C_12_H_16_O_12_	+	+	-	+	-	-	-
M41 *	8.06	C_20_H_17_O_5_	337.10815	337.10739	12.5	1.008	337.11(100),252.04(14),265.05(11),319.10(9)	M+O	-	-	-	+	+	-	-
M42	8.13	C_20_H_17_O_5_	337.10815	337.10742	12.5	1.097	337.11(100),252.04(14),265.05(12),319.10(5)	M+O	+	-	-	-	-	-	-
M43	8.15	C_20_H_15_O_6_	351.08741	351.08661	13.5	0.841	252.04(29),351.09(7),266.06(2)	M-H_2_+O_2_	+	-	-	-	-	-	-
M44	6.10	C_20_H_19_O_3_	307.13397	307.13010	11.5	−1.273	307.10(100),162.02(10),252.04(7),236.14(4)	M-O+H_2_	-	-	-	+	+	-	-
M45	8.16	C_19_H_15_O_4_	307.09758	307.09665	12.5	0.536	237.05(0.44),135.01(0.40),253.05(0.38),267.06(0.37),	M-CH_2_	-	-	-	-	+	-	+
M46	8.18	C_20_H_17_O_8_S	417.06496	417.06415	12.5	0.684	113.02(49),417.07(38),79.96(29),337.11(25),319.10(20),96.96(19)	M+SO_4_	-	-	-	+	-	-	-
M47	8.21	C_26_H_25_O_10_	497.14532	497.14441	14.5	0.375	321.11(100),113.02(78),497.14(55),175.02(29)	M+C_6_H_8_O_6_	+	+	+	+	-	-	-
M48	8.21	C_27_H_27_O_11_	527.15583	527.15454	14.5	−0.470	336.10(100),113.02(50),351.12(46),175.02(23),527.15(15)	M+C_6_H_8_O_6_+CH_2_O	-	-	-	+	-	-	-
M49 *	8.22	C_20_H_19_O_5_	339.12380	339.12277	11.5	0.206	339.12(100),252.04(5),267.06(2),237.05(0.34)	M+H_2_O	-	-	-	+	+	-	-
M50	8.46	C_20_H_19_O_3_	307.13397	307.13129	11.5	−4.855	307.12(100),263.13(63),219.14(72)	M-O+H_2_	-	-	-	+	+	-	-
M51	8.52	C_26_H_25_O_10_	497.14532	497.14456	14.5	0.436	321.11(73),113.02(40),175.02(18),497.14(8),265.05(5)	M+C_6_H_8_O_6_	-	-	+	+	-	-	-
M52	8.66	C_26_H_25_O_10_	497.14532	497.14441	14.5	0.255	321.11(100),113.02(55),175.02(25),497.14(10),265.05(7)	M+C_6_H_8_O_6_	-	-	-	+	-	-	-
M53	8.66	C_20_H_17_O_11_S_2_	497.02175	497.02170	12.5	2.054	321.11(100),113.02(58),497.15(13),95.01(7)	M+SO_3_+SO_4_	-	-	-	+	-	-	-
M54	8.75	C_20_H_17_O_5_	337.10815	337.10742	12.5	1.097	337.11(100),265.05(47),252.04(11)	M+O	-	-	-	+	+	-	-
M55	8.95	C_19_H_15_O_4_	307.09758	307.09689	12.5	1.415	223.04(0.26),305.08(0.21),221.06(0.17),238.06(0.15),	M-CH_2_	-	-	-	-	-	-	+
M56 *	9.08	C_20_H_17_O_7_S	401.07005	401.06885	12.5	−0.249	321.11(100),401.07(59),121.03(7),79.96(6)	M+SO_3_	-	-	+	-	-	-	-
M57	8.90	C_25_H_25_O_9_	469.15043	469.15167	13.5	4.138	469.21(100),113.02(51),293.17(47),275.16(38),175.02(16),401.07(13)	M+C_6_H_8_O_6_-CO	+	-	-	-	-	-	-
M58	9.18	C_20_H_17_O_7_S	401.07003	407.06934	12.5	0.972	321.11(100),401.07(64.82),121.03(30.52),79.96(7.54)	M+SO_3_	+	+	+	+	+	+	+
M59	9.24	C_27_H_27_O_10_	511.16133	511.15991	14.5	0.072	511.16(100),277.05(84),265.05(34),320.10(22),321.11(10),113.02(1)	M+C_6_H_8_O_6_+CH_2_	-	-	-	+	-	-	-
M60	9.33	C_20_H_17_O_7_S	401.07003	401.06888	12.5	−0.175	79.96(39.99),321.11(32.15),401.08(27.66),96.96(10.39)	M+SO_3_	-	-	-	-	+	-	-
M61	9.45	C_20_H_19_O_5_	339.12380	339.12234	11.5	−1.151	309.11(100),339.12(51),253.05(22)	M+H_2_O	-	-	-	-	+	-	-
M62	9.65	C_21_H_19_O_5_	351.12380	351.12268	12.5	−0.057	319.10(54),351.12(52),321.11(18),271.21(13),307.19(10)	M+CH_2_O	-	-	-	+	+	-	+
M63	9.73	C_20_H_17_O_5_	337.10815	337.10690	12.5	0.207	337.11(100),265.14(14),293.12(9),309.11(4)	M+O	-	-	-	+	+	-	-
M64	10.40	C_20_H_17_O_5_	337.10815	337.10703	12.5	−0.060	293.17(100),337.10(42),137.02(20)	M+O	-	-	-	+	-	-	-
M65	10.61	C_20_H_17_O_11_S_2_	497.02175	497.02332	12.5	3.040	497.24(54),113.02(27),321.21(257),95.01(5)	M+SO_3_+SO_4_	-	+	-	-	-	-	-
M66	11.15	C_20_H_15_O_5_	335.09250	335.09146	13.5	0.179	317.21(21),299.20(11),291.23(10),335.09(6)	M-H_2_+O	-	-	-	-	+	-	-
M67	12.67	C_20_H_15_O_4_	320.10431	319.09653	13.5	0.140	319.10(100),116.93(6),304.07(6),	M-H_2_	-	-	-	+	-	-	-
M68	15.44	C_20_H_17_O_10_S	449.05475	449.05615	12.5	−0.632	116.93(100),151.02(47),379.21(9),96.96(7)	M+SO_3_+O_3_	-	-	-	-	-	+	-
M69	17.35	C_19_H_19_O_3_	295.13397	295.13120	10.5	−3.188	295.23(100),134.89(5),113.10(5),179.14(2),254.99(2)	M-CO+H_2_	-	+	-	-	-	-	-
M70	19.06	C_19_H_19_O_3_	295.13397	295.13028	10.5	−4.950	295.23(65),265.26(3),120.13(3)	M-CO+H_2_	-	-	+	-	+	-	-
M71	23.06	C_25_H_24_O_7_NS	482.12790	482.12555	14.5	−2.591	129.97(23),412.21(20),116.93(11),482.13(6)	M+C_5_H_7_NO_3_S	-	-	+	-	-	-	-
M72	23.09	C_20_H_17_O_10_S_2_	481.02685	481.02747	12.5	3.546	412.21(25),129.97(22),481.30(4),96.96(2),400.74(2)	M+SO_3_+SO_3_	-	+	-	-	-	-	-

Note: t_R_: retention time; PM: methanol-precipitated plasma samples; PA: acetonitrile-precipitated plasma samples; PS: plasma samples prepared by SPE; U: urine samples; F: fecal samples; L: liver samples; LM: liver microsome samples; +: detected; -: undetected; *: components in Table 3

**Table 3:** Ingredient screening list.

Compound	Structural Formula	Reaction	Bioavailability Score	Drug-Likeness (Yes)	Number of Targets
M0	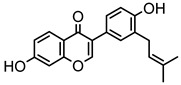	Neobavaisoflavone	0.55	5	33
N1	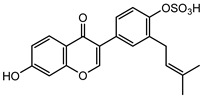	Sulfation	0.56	5	35
N2	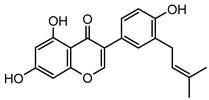	Hydroxylation	0.55	5	40
N3	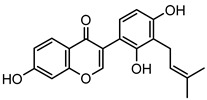	Hydroxylation	0.55	5	33
N4	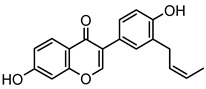	Demethylation	0.55	5	36
N5	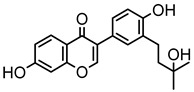	Hydration	0.55	5	39
N6	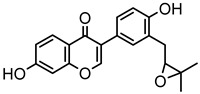	Epoxidation	0.55	5	25
N7	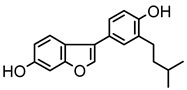	Decarbonylation Hydrogenation	0.55	4	66
N8	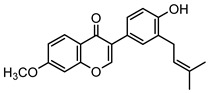	Methylation	0.55	5	99

## Data Availability

Not applicable.
